# HMGB1 as a drug target in staphylococcal pneumonia

**DOI:** 10.1186/cc13810

**Published:** 2014-03-31

**Authors:** Mitchell P Fink

**Affiliations:** 1 Departments of Surgery and Anesthesiology, David Geffen School of Medicine at the University of California Los Angeles, Los Angeles, CA 90095, USA

## Abstract

High mobility group box (HMGB)1 is a small DNA-binding protein. In the nucleus, HMGB1 plays a role in gene expression and DNA replication. When it is released or secreted into the extracellular milieu, HMGB1 functions as a pro-inflammatory cytokine-like mediator. Recently reported data support the view that treatment with a neutralizing anti-HMGB1 antibody ameliorated pulmonary damage in a murine model of pneumonia caused by a pathogenic strain of *Staphylococcus aureus*. These findings suggest that HMGB1 may be an important drug target as scientists, clinical investigators and pharmaceutical companies seek to develop better agents for the treatment of staphylococcal pneumonia. Unfortunately, however, encouraging results from murine models of human disease often fail to translate into positive findings in clinical trials. Thus, before moving from pre-clinical into clinical studies, it may be prudent to validate and extend the recent experimental findings by carrying out additional studies, using a large animal model of pneumonia.

## 

In a recent article in *Critical Care*, Achouiti and co-workers [1] from the Academic Medical Center in Amsterdam explored the role of high mobility group box (HMGB)1 in an animal model of pneumonia caused by a methicillin-resistant, highly pathogenic strain of *Staphylococcus aureus*. In order to better understand the implications of these findings, it is useful to briefly review the ‘HMGB1 as inflammatory mediator’ story.

HMGB1 was originally identified by Shooter and colleagues [2] as a relatively small, highly charged non-histone DNA-binding protein. About 25 years later, Wang and colleagues [3] identified HMGB1 as a late-acting mediator of lipopolysaccharide-induced lethality in mice. This important paper opened up a fresh avenue of research in immunology, one that led to the recognition that certain endogenous molecules, which can be passively released by stressed or necrotic cells or, in some cases, actively secreted by immunostimulated macrophages and certain other cell types, are capable of activating the innate immune system and initiating or propagating inflammation. These molecules are now collectively referred to as ‘damage-associated molecular patterns’ (DAMPs). In addition to HMGB1, other DAMPs include uric acid, heat shock proteins, adenosine triphosphate, and partially degraded forms of hyaluronic acid [4].

HMGB1 is a multifunctional protein. In the nucleus, it participates in DNA replication and the regulation of gene expression [5]. When it is released or secreted into the extracellular milieu, HMGB1 can act as a chemokine (that is, a chemoattractant for motile leukocytes) or a cytokine (that is, an immune system mediator) to promote the secretion of other cytokines, such as tumor necrosis factor, IL-1, and IL-6, by macrophages and other cell types. Whether extracellular HMGB1 acts as a chemokine, cytokine or neither depends upon the redox state of three critical cysteine residues (C23, C45 and C106) in the protein [6,7]. In order for HMGB1 to function as a chemokine, the thiol moieties in all three of these cysteine residues must exist in their fully reduced form. In order to function as a cytokine, two of the cysteine residues, C23 and C45, must form a disulfide bond, whereas the third cysteine residue, C106, must remain fully reduced. If the cysteine residues are fully oxidized to the sulfonate form, then both the chemokine and cytokine functions are abrogated. In its role as a cytokine, HMGB1-dependent signaling is initiated primarily by activation of Toll-like receptor (TLR)4 and the receptor for advanced glycation endproducts (RAGE). In its role as a chemokine, HMGB1 forms a heterocomplex with the chemokine CXCL12 and initiates signaling via activation of the receptor CXCR4 [8].

Abundant evidence supports the view that HMGB1 is an important pathological mediator in animal models of several human diseases, including sepsis [9], hemorrhagic shock [10], and acute respiratory distress syndrome (ARDS) [11]. At present, ironclad evidence that HMGB1 is an important mediator of disease in humans is still lacking, although elevated circulating levels of HMGB1 have been detected in patients with sepsis [12], ARDS [13] or rheumatoid arthritis [14] and high levels of HMGB1 have been found in samples of synovial fluid from patients with rheumatoid arthritis [15].

Achouiti and colleagues [1] employed a murine model of sublethal pneumonia caused by a virulent strain of methicillin-resistant *S. aureus* (MRSA). Using wild-type mice as well as TLR4 and RAGE ‘knock-out’ (KO) mice, Achouiti and colleagues showed that *S. aureus* pneumonia was associated with HMGB1 release into bronchoalveolar lavage fluid. Treatment with a neutralizing anti-HMGB1 antibody ameliorated pulmonary damage. RAGE KO mice were partially protected from lung damage caused by *S. aureus* pneumonia, whereas TLR4 KO mice were not protected at all.

The data obtained by Achouiti and colleagues suggest that HMGB1 might be a viable drug target in patients with pneumonia caused by *S. aureus*, but the study conducted by Achouiti and colleagues has some limitations. First, the authors studied the role of HMGB1 in a *S. aureus* pneumonia model in the absence of appropriate antimicrobial chemotherapy. It is impossible to know whether administration of an effective antibiotic would have affected the results. Second, the mice were treated with the neutralizing anti-HMGB1 antibody (or the control antibody) prior to the onset of infection. In the clinical setting, treatment almost always would start after the onset of infection. Third, concerns have been raised about the predictive utility of murine models of sepsis [16]. These concerns stem from recent data showing that changes in gene expression in acutely endotoxemic mice are quite distinct from those observed in acutely endotoxemic human volunteers [17]. Furthermore, most studies using mice are conducted with the animals maintained at ‘room temperature’ (20 to 23°C). Housing laboratory mice under more physiological conditions (for the animals) might lead to results that are more applicable to the clinical situation [18]. Carrying out studies using a clinically relevant large animal model of MRSA pneumonia, such as the one described by Rehberg and colleagues [19], might be another way to validate the findings obtained in mice.

### Conclusion

Staphylococcal pneumonia is an important clinical problem [20,21]. There clearly is a pressing, unmet clinical need for improved therapeutics for staphylococcal (particularly MRSA) pneumonia. Developing drugs that target HMGB1 may be one way to tackle this problem, although moving from the encouraging findings reported by Achouiti and co-workers to the registration of a new medicine will be an arduous task.

### Abbreviations

ARDS: Acute respiratory distress syndrome; DAMP: Damage-associated molecular pattern; HMGB: High mobility group box; IL: Interleukin; KO: Knock-out; MRSA: Methicillin-resistant *S. aureus*; RAGE: Receptor for advanced glycation endproducts; TLR: Toll-like receptor.

### Competing interests

The authors declare that they have no competing interests.
